# Ultrasmall Conjugated Polymer Nanoparticles with High Specificity for Targeted Cancer Cell Imaging

**DOI:** 10.1002/advs.201600407

**Published:** 2017-05-01

**Authors:** Guangxue Feng, Jie Liu, Rongrong Liu, Duo Mao, Nikodem Tomczak, Bin Liu

**Affiliations:** ^1^ Department of Chemical and Biomolecular Engineering National University of Singapore 4 Engineering Drive 4 117585 Singapore; ^2^ Institute of Materials Research and Engineering (IMRE) 2 Fusionopolis Way Innovis 136834 Singapore

**Keywords:** cancer targeting, conjugated polymers, fluorescence imaging, organic nanoparticles

## Abstract

Fluorescent and biocompatible organic nanoparticles have attracted great interest in cancer detection and imaging, but the nonspecific cellular uptake has limited the detection specificity and sensitivity. Herein, the authors report the ultrasmall conjugated polymer nanoparticles (CPNs) with bright far‐red/near‐infrared emission for targeted cancer imaging with high specificity. The sizes of the ultrasmall CPNs are around 6 nm (CPN6), while large CPNs show sizes around 30 nm (CPN30). Moreover, CPN6 exhibits largely improved fluorescence quantum yield (η) of 41% than CPN30 (25%). Benefiting from the ultrasmall size, bare CPN6 shows largely suppressed nonspecific cellular uptake as compared to CPN30, while cyclic arginine‐glycine‐aspartic acid (cRGD) functionalized CPN6 (cRGD‐CPN6) possesses excellent selectivity toward *α_v_β*_3_ integrin overexpressed MDA‐MB‐231 cells over other cells in cell mixtures. The faster body clearance of CPN6 over CPN30 indicates its greater potentials as a noninvasive nanoprobe for in vivo and practical applications.

## 1 Introduction

The detection and imaging of specific cancer cells with high selectivity and sensitivity is of great importance for cancer diagnosis and therapeutics.^1^, ^2^ Fluorescence imaging as a safe, cost‐effective modality with high temporal and spatial resolution appears to be one of the indispensable platforms.^3^, ^4^ In particularly, fluorescent nanoparticles (NPs) covalently linked with biorecognition ligands have shown great merits in cancer diagnosis and therapy.^5^, ^6^, ^7^, ^8^ As compared to molecular dye‐ligand conjugates, the multiple targeting ligands presented on NP surfaces allow multivalent recognition and binding to receptors on cell membrane, resulting in higher binding affinity and much improved selectivity toward targeted cells.^9^, ^10^, ^11^ With the rapid advances of nanotechnology, versatile NP systems including semiconducting quantum dots (QDs), gold NPs, and dye encapsulated NPs, etc., have been developed.^12^, ^13^, ^14^, ^15^, ^16^ Despite the fact that NPs with varied sizes have been designed for different biomedical applications,^17^, ^18^, ^19^ there has been limited success in highly selective cancer targeting, and only a few examples for inorganic gold NPs were reported.^16^, ^20^ This is attributed to the precise size control of inorganic NPs to be smaller than 10 nm, as NPs with sizes ranging from 20 to 200 nm are able to be nonspecific internalized into cells which significantly compromises the detection selectivity.^21^, ^22^, ^23^, ^24^, ^25^ However, inorganic NPs inherently born with heavy metal components are difficult to decompose, and they may also have potential toxicity.^26^ As a consequence, for selective cancer detection with high sensitivity in a complicated environment where different cell lines are present, biocompatible organic NPs with sub‐10 nm size level are highly desirable.

Conjugated polymers (CPs) as a novel class of fluorescent molecules have attracted great interest in bioimaging and biomedical applications due to their light harvesting properties, high extinction coefficients, good photostability, and controllable emission from ultraviolet to far‐red/near‐infrared (FR/NIR) (>650 nm) regions.^27^, ^28^, ^29^, ^30^ In particular, the development of CP nanoparticles (CPNs) with excellent water dispersibility, amendable surface chemistry, and versatile functions has shown their unique merits in bioimaging.^31^, ^32^, ^33^, ^34^ Recently, the design of CPs or CPNs with FR/NIR emission has attracted great research interests due to the low biological autofluorescence and high tissue penetration depth in the FR/NIR region.^35^, ^36^, ^37^ Despite the enormous efforts in the development of FR/NIR CPNs,^38^, ^39^, ^40^ they generally show lower fluorescence quantum yields as compared to those in the visible region due to the strong π‐stacking and intramolecular charge transfer in aqueous media. Through the introduction of narrow‐band‐gap moieties into CP backbones, we have designed new FR/NIR emissive CPs with much improved fluorescence quantum yield in the NPs.^39^, ^40^ However, due to the relatively large particle size, these NPs showed nonspecific cellular uptake toward nontargeted cells, which largely compromises the detection sensitivity.^40^, ^41^, ^42^ So far, great efforts have been made on the design of CPNs with varied sizes ranging from 15 nm to 2 µm;^31^, ^42^, ^43^, ^44^, ^45^, ^46^ however, stable CPNs with sub 10 nm size and targeting ability remains challenging.

In this contribution, we report the design and synthesis of CPNs with ultrasmall size of ≈6 nm (CPN6) and bright FR/NIR emission for in vitro targeted cancer imaging with high sensitivity and specificity in cell mixtures. CPNs with ≈30 nm (CPN30) size were also synthesized for comparison. The size, morphology, optical properties, photostability, and biocompatibility of both CPNs are characterized. The evaluation of CPNs in selective cancer cell detection includes the study of nonspecific cellular imaging, peptide decorated CPNs for targeted fluorescence imaging, and cellular uptake mechanism. The successful demonstration of cyclic arginine‐glycine‐aspartic acid (cRGD) decorated CPN6 (cRGD‐CPN6) for selective detection toward *α_v_β*_3_ integrin overexpressed MDA‐MB‐231 cells over other cells clearly reveal the superior sensitivity and specificity of cRGD‐CPN6 for recognition of integrin in complicated and more realistic environment where different cell lines present simultaneously. In vivo experiments further revealed the fast body clearance of CPN6, making them noninvasive for practical applications. This demonstration of ultrasmall CPNs should open new opportunities for the development of organic nanomaterials for cancer diagnosis and therapy, particularly for immunostaining or potential direct cell labeling in vivo.

## 2 Results and Discussion

**Figure 1:**
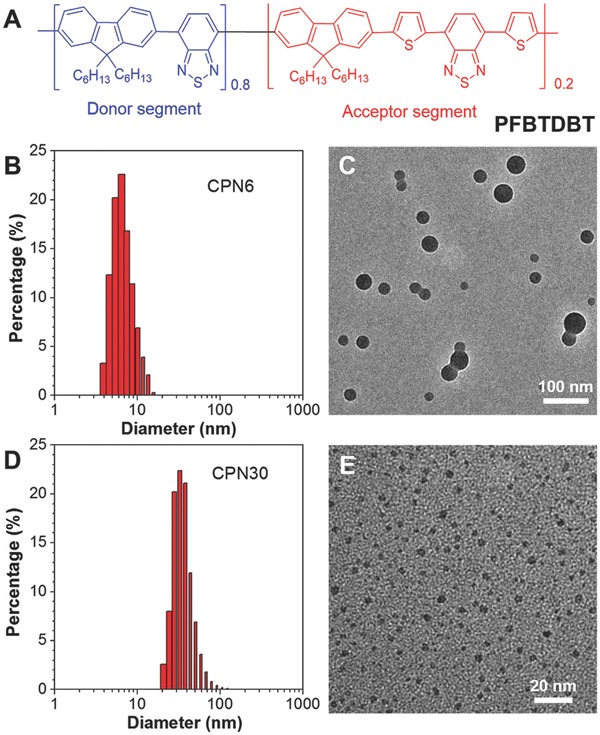
A) Chemical structure of PFBTDBT. B) Size distribution and C) TEM image of CPN30. D) Size distribution and E) TEM image of CPN6.

Poly[(9,9‐dihexylfluorene)‐*co*‐2,1,3‐benzothiadiazole‐*co*‐4,7‐di(thiophen‐2‐yl)‐2,1,3‐benzothiadiazole] (PFBTDBT) (*M*_n_ = 25 300, polydispersity index (PDI) = 1.8) (**Figure** **1**A) with FR/NIR emission was synthesized according to our previous report.^40^ Lowering the narrow band gap monomer 4,7‐modi(thiophen‐2‐yl)‐2,1,3‐benzothiadiazole contents helps suppress the aggregation and concentration caused quenching, leading to improved FR/NIR emission in the aggregated state. Maleimide functionalized block copolymer, 1,2‐distearoyl‐*sn*‐glycero‐3‐phosphoethanolamine‐*N*‐[maleimide(polyethylene glycol)‐2000] (DSPE‐PEG‐Mal), was selected as the encapsulation matrix for CPN fabrication, due to its excellent encapsulation performance and biocompatibility.^31^ CPN30 was fabricated using a modified nanoprecipitation method as previously reported,^31^, ^47^ where a homogenous tetrahydrofuran (THF) solution of PFBTDBT and DSPE‐PEG‐Mal was added into MilliQ water under ultrasound sonication. During the mixing and sonication, DSPE‐PEG‐Mal will intertwine with PFBTDBT to form the stable CPNs. Laser light scattering (LLS) (Figure 1B) reveals that CPN30 shows an average hydrodynamic diameter of ≈32 nm with a low PDI of ≈0.1.

To realize the ultrasmall size for CPN6, a different approach was applied. DSPE‐PEG‐Mal was dissolved in aqueous solution (1 mg mL^−1^) and used as the surfactant, where pure THF solution of PFBTDBT was added into DSPE‐PEG‐Mal aqueous solution at a volume ratio of 1:10 under water batch sonication. The diffusion of PFBTDBT chains from soluble THF phase to insoluble aqueous phase naturally leads to the polymer aggregates as the hydrophobic core, while the DSPE‐PEG‐Mal polymers wrap theses cores and stabilize them from further aggregation. The low PFBTDBT loading concentration (0.1 mg mL^−1^) was used to prevent the formation of large aggregates during the encapsulation process. After prolonged sonication for 30 min, the mixture was dialyzed against water to remove THF and excess DSPE‐PEG‐Mal, and the CPNs were collected to show sizes of ≈6 nm as revealed by LLS (Figure 1D). It should be noted that upon increasing the loading concentration, the sizes of the obtained CPNs also increase in a controllable manner (Figure S1, Supporting Information). High‐resolution transmission electron microscopy (HR‐TEM) was further used to study their morphology, where both CPN30 and CPN6 showed distinguishable spherical shapes with size around 30 and 6 nm, respectively (Figure 1C,E). In addition, CPN6 exhibited more uniform distribution in the dry state, indicating excellent control of size distribution for the ultrasmall CPNs. Moreover, both CPNs exhibited similar surface charge, where the zeta potentials for CPN6 and CPN30 are −17.7 and −15.4 mV, respectively.

**Figure 2:**
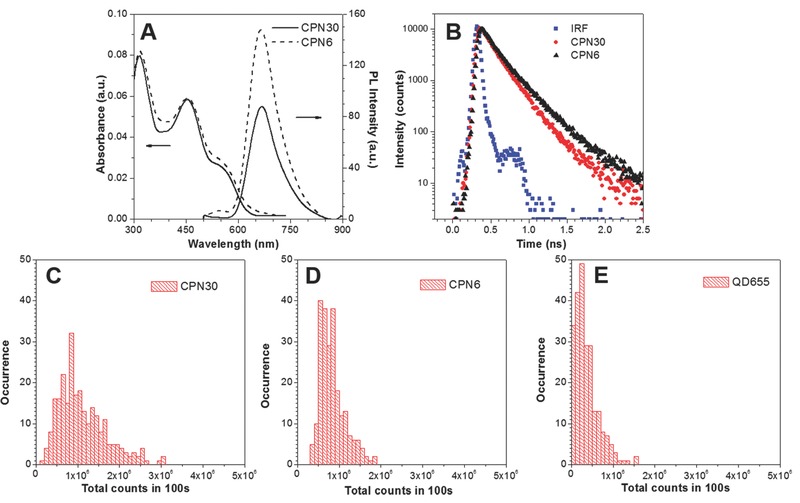
A) UV–vis absorption and emission spectra, and B) fluorescence decay at 670 nm of CPN6 and CPN30 in water, IRF refers to instrumental response function. C–E) Histograms of the total photon counts emitted during 100 s by C) CPN30, D) CPN6, and E) QD655.

**Figure** **2**A shows the UV–vis absorption and emission spectra of CPN6 and CPN30 in aqueous media. They have similar absorption and emission spectra, where two absorption peaks are centered at 325 and 465 nm, and the emission maximum is localized at 670 nm. Such a large Stokes shift of over 200 nm minimizes the excitation light source interference for bioimaging. At the same absorbance, CPN6 shows much brighter fluorescence than CPN30, where the fluorescence quantum yields (η) for CPN6 and CPN30 are ≈41% ±  2% and 25% ± 1%, respectively, measured using 4‐(dicyanomethylene)‐2‐methyl‐6‐(*p*‐dimethylaminostyryl)‐4 H‐pyran in methanol (η = 43%) as the standard. Such a remarkable difference in η should be due to the much smaller size of CPN6 and lower loading amount of PFBTDBT as compared to CPN30, which helps to suppress the fluorescence quenching due to the aggregation of PFBTDBT chains. It should be noted that the presence of PEG shell could interfere the direct interactions between PFBTDBT and water or oxygen molecules, which should also contribute to the high brightness of CPN6.^48^

To better understand the η enhancement for CPN6, we further investigated the fluorescence lifetimes (τ) for both CPNs. τ and η are related to the radiative decay rate (*k*_r_) and nonradiative decay rate (*k*_nr_) through the following equations: η = *k*_r_/(*k*_r_ + *k*_nr_) and τ = 1/(*k*_r_ + *k*_nr_). *k*_r_ is an intrinsic property of a fluorophore, which in general is kept constant. Therefore, τ and η are changing in the same direction and mainly affected by the nonradiative pathway. Figure 2B shows the fluorescence decay curves for CPN6 and CPN30, where CPN6 exhibits elongated fluorescence lifetimes of 1.53 ns as compared to CPN30 (1.38 ns) (Table S1, Supporting Information). The lifetime difference clearly indicates the better suppression of the nonradiative decay pathways of the PFBTDBT inside CPN6.

The brightness of CPN6 and CPN30 at individual NP level was then investigated using single NP fluorescence wide‐field microscopy. Recognized with high brightness, commercially available semiconductor quantum dots (QD655) with similar emission maximum as PFBTDBT NPs was selected as the benchmark. The fluorescence of each CPN/QD655 is traced over 100 s by 1000 consecutive frames (Figure S2, Supporting Information), and the brightness of each CPN is collected by integrating the emission for the 1000 consecutive frames. Figure 2C–E showed the histograms of the total photon numbers emitted by CPN30, CPN6, and QD655 within 100 s, respectively. The total number of photons on average emitted by each CPN6 (8.3 × 10^5^ counts) is slightly smaller than that of CPN30 (10.9 × 10^5^ counts), while both are over two times higher than that of QD655 (4.1 × 10^5^ counts), indicating the high brightness of our CPNs in the FR/NIR region. As each CPN6 has a smaller size than CPN30, it should possess less number of PFBTDBT chains per NP, and the similar photon counts further prove the higher fluorescence η of CPN6. Moreover, CPN6 exhibited much narrower brightness distribution as compared to that of CPN30, indicating more uniformly distributed PFBTDBT polymers in each CPN6.

**Figure 3:**
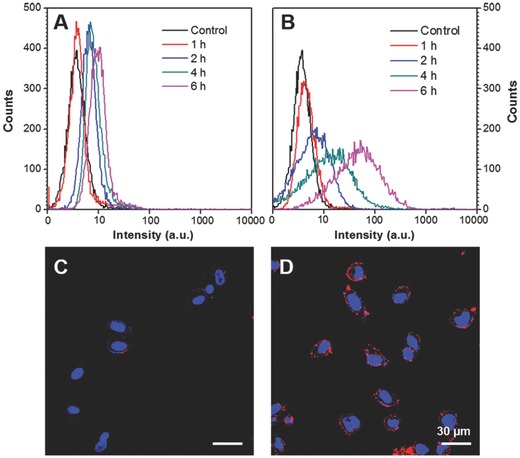
Flow cytometry histograms of MDA‐MB‐231 cells after incubation with A) CPN6 and B) CPN30 for different time. Confocal images of MDA‐MB‐231 cells after 6 h incubation with C) CPN6 and D) CPN30 at the same PFBTDBT concentration (0.01 mg mL^−1^). Red channel: *E*_x_ = 543 nm, *E*_m_ = above 605 nm; blue channel: *E*_x_ = 405 nm, *E*_m_ = 430–470 nm.

The cellular uptake performance of CPN6 and CPN30 was then studied by flow cytometry and confocal laser scanning microscopy (CLSM). MDA‐MB‐231 breast cancer cells were selected as the model cell lines, which were incubated with CPN6 and CPN30 suspension (based on PFBTDBT concentration of 0.01 mg mL^−1^) in cell culture medium for different time. **Figure** **3**A,B shows the fluorescence profiles of MDA‐MB‐231 cells after these treatments. Upon increasing incubation time, CPN6 treated cells still show very low brightness profiles with narrow distribution similar to controlled blank cells, indicating poor cellular uptake of CPN6. As for CPN30, although the fluorescence inside cells remains dark after 1 h incubation, it quickly increases at prolonged incubation time. After 6 h incubation, a distinguishable fluorescence profile was clearly observed, indicating effective cellular uptake of CPN30. Figure 3C,D shows the CLSM images of MDA‐MB‐231 cells after 6 h treatment with CPN6 and CPN30, respectively. Almost no red fluorescence can be detected for CPN6 treated cells while bright red emission from CPN30 can be clearly observed on the cell membrane and inside cell cytoplasm, which is in consistent with the flow cytometry results. The results clearly indicate that the nonspecific cellular uptake is largely suppressed for CPN6. Along with the presence of PEG at surface which also helps to reduce the nonspecific cellular uptake,^49^, ^50^, ^51^, ^52^, ^53^ the further functionalized CPN6 is expected to exhibit good selectivity in targeted cancer cell imaging.

**Figure 4:**
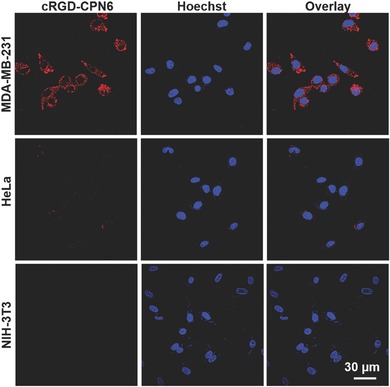
A) CLSM images of MDA‐MB‐231 (upper row), HeLa (middle row), and NIH‐3T3 cell lines treated by cRGD‐CPN6 for 6 h, the nucleus was stained by Hoechst. Red channel: *E*_x_ = 543 nm, *E*_m_ = above 605 nm; blue channel: *E*_x_ = 405 nm, *E*_m_ = 430–470 nm.

Inspired by the largely suppressed nonspecific cellular internalization of CPN6, we functionalized it with targeting ligands for selective detection and imaging of cancer cells. Cyclic arginine‐glycine‐aspartic acid (cRGD) tripeptide that can selectively bind to *α_v_β*_3_ integrin was conjugated to CPN6 surface to yield cRGD‐CPN6. MDA‐MB‐231 cells with *α_v_β*_3_ overexpression on cell membrane were selected as the target, while HeLa cancer cells and NIH‐3T3 normal cells with low integrin expression were used as the negative control.^54^, ^55^ After 6 h incubation, bright red fluorescence from cRGD‐CPN6 in the cytoplasm was observed in MDA‐MB‐231 cells, while the red fluorescence remain silent in HeLa and NIH‐3T3 cells, indicating that cRGD‐CPN6 is selectively internalized into the targeted cells (**Figure** **4**). The high selectivity and specificity is further confirmed by flow cytometry (Figure S3, Supporting Information), where the average fluorescence intensity of MDA‐MB‐231 cells is 28‐fold higher than that of NIH‐3T3 cells.

**Figure 5:**
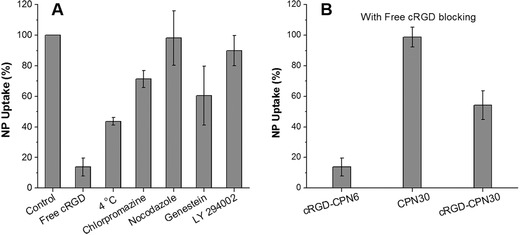
A) Cell uptake efficiencies of cRGD‐CPN6 toward MDA‐MB‐231 cells pretreated with different endocytosis inhibitors. B) Cell uptake efficiencies of cRGD‐CPN6, CPN30, and cRGD‐CPN30 toward MDA‐MB‐231 cells preblocked with free cRGD. The uptake efficiency without inhibitor treatment or cRGD blocking is arbitrarily set to 100%.

To gain more insights into the cellular uptake mechanism of cRGD‐CPN6, we evaluated the effects of different endocytosis inhibitors on the cellular uptake of cRGD‐CPN6, where MDA‐MB‐231 cells were treated with different inhibitors prior to cRGD‐CPN6 incubation.^56^, ^57^ As shown in **Figure** **5**A, a moderate decrease in cellular uptake is found for cells pretreated with chlorpromazine or genistein or incubated at 4 °C, while no obvious change was observed upon pretreatment with nocodazole. The reduced cellular uptake at 4 °C clearly indicates that cellular uptake of cRGD‐CPN6 is energy dependent. As chlorpromazine can inhibit clathrin‐mediated endocytosis, nocodazole is caveolae‐mediated endocytosis inhibitor, and genistein can inhibit both clathrin‐ and caveolae‐mediated endocytosis,^56^, ^57^ these results clearly indicate that cRGD‐CPN6 enters MDA‐MB‐231 cells mainly through energy dependent clathrin‐mediated endocytosis pathway. Moreover, the pretreatment of LY294002 hardly affected cRGD‐CPN6 cellular uptake, revealing that micropinocytosis is not involved. The pretreatment of free cRGD could largely inhibit the uptake of cRGD‐CPN6 toward MDA‐MB‐231 cells, indicating that the cRGD‐CPN6 enters MDA‐MB‐231 cells mainly through integrin receptor mediated clathrin endocytosis pathway. However, pretreatment with free cRGD can only partially block cellular uptake of cRGD‐CPN30 while the cellular uptake of CPN30 is not affected by free cRGD blocking (Figure 5B). Collectively, the 30 nm NPs can enter cells with or without receptor recognition and mediation, while 6 nm NPs enter cell mainly through receptor mediated endocytosis, and hence CPN6 is able to provide higher sensitivity and selectivity in cancer cell detection.

**Figure 6:**
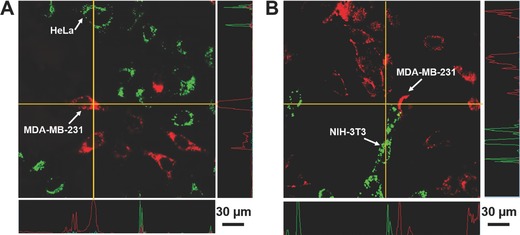
CLSM images of cRGD‐CPN6 treated A) HeLa and MDA‐MB‐231 cell mixture and B) NIH‐3T3 and MDA‐MB‐231 cell mixture. HeLa and NIH‐3T3 cells were pretreated with CellTracker 540 to be green emissive (2 × 10^−9^ m, 2 h) before mixing with the same amount of MDA‐MB‐231 cells. Green channel: *E*_x_ = 488 nm, *E*_m_ = 505–525 nm; red channel: *E*_x_ = 543 nm, *E*_m_ = above 605 nm.

We further demonstrated the excellent selectivity of cRGD‐CPN6 in a more complicated environment where multiple cell lines simultaneously exist in the same incubation chamber before CPN treatment. To achieve this, MDA‐MB‐231 and HeLa or NIH‐3T3 cells were mixed together and cocultured until confluence was reached. HeLa and NIH‐3T3 cells were pretreated with CellTracker540 to become green emissive, which can be easily differentiated from MDA‐MB‐231 cells in the cell mixture. CellTracker540 is a commercial green emissive product provided by Lunimicell for cell tracing with high cellular retention and minimal cell activity disturbance.^47^, ^58^ The cell mixture was then incubated with cRGD‐CPN6 at PFBTDBT concentration of 0.01 mg mL^−1^ for 6 h. As shown in **Figure** **6**, the green emissive HeLa cells and NIH‐3T3 cells are clearly distinguishable from the red fluorescent MDA‐MB‐231 cells. In addition, there is no interference and crosstalk between green and red fluorescence in each individual cell, indicating that cRGD‐CPN6 can only be internalized into targeted MDA‐MB‐231 cells even in the presence of other cells. This further strengthens the excellent sensitivity and specificity of CPN6. This is significantly different from that of CPN30, which exhibited a remarkable uptake toward untargeted control cells.^40^

The ultrasmall CPNs also exhibit excellent stability. Upon incubation of CPN6 in serum solution or phosphate buffered saline (PBS) buffers with increased salt concentrations, its sizes as measured by LLS do not exhibit obvious changes (Figure S4, Supporting Information). In addition, its emission intensity showed negligible changes after 5 d incubation in PBS buffer (Figure S5, Supporting Information), which further indicate the excellent colloidal stability of CPN6. Under continuous laser scanning, the brightness of CPNs inside cells remains high and stable. Semiquantitative analysis reveals that the emission intensity of CPNs inside cells is over 90% of its original values after 10 min continuous scanning (Figure S6, Supporting Information), which is similar to QD655 with ultrahigh photostability. It is much better than fluorescein isothiocyanate which exhibits over 50% signal loss under the same experimental conditions, indicating excellent photostability of the CPNs. In addition, the viabilities of cells after incubation with the CPN6, CPN30, cRGD‐CPN6 were tested via methylthiazolyldiphenyltetrazolium (MTT) assay (Figure S7, Supporting Information), which revealed a high cell viability over 90% even after 48 h incubation with much concentrated CPNs (at PFBTDBT concentration of 0.1 mg mL^−1^), demonstrating the low cytotoxicity of the CPNs.

**Figure 7:**
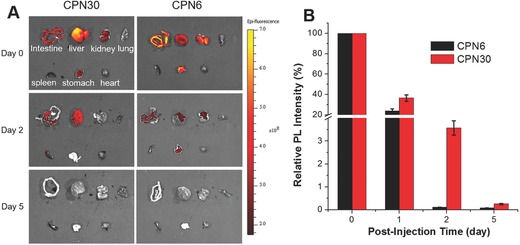
A) Ex vivo fluorescence images of organs at various time postadministration of CPNs. B) Relative fluorescence intensity of CPNs in the blood at various time postadministration.

As an imaging contrast agent, fast body clearance after injection is highly beneficial for noninvasive diagnosis. We further performed the in vivo experiments to study the biodistribution and clearance of both CPNs using BALB/c nude mice. The CPNs were intravenously injected into mice via tail vein. After designated time points, the mouse blood were collected and the fluorescence intensity in blood was recorded; and then the mice were sacrificed and the organs including intestine, liver, kidney, lung, spleen stomach, and heart were isolated and imaged. As shown in **Figure** **7**A, at 2 h postadministration, intense fluorescence from CPN30 is mainly observed from liver tissue, which is much brighter than intestine and kidney, while other organs including lung, spleen, and heart exhibit negligible fluorescence. As for CPN6, the accumulation at intestine, kidney, stomach, and liver was observed, where liver gives the lowest fluorescence. It is also noted that the signals from these organs decreased as time elapsed, where no fluorescence can be detected at day 5 postadministration. Concluded from Figure 7A, CPN30 is mainly enriched in the reticuloendothelial system organs, and its clearance is largely through biliary pathway, which shows increased uptake and quick removal of NPs in liver. On the other hand, CPN6 is excreted from mouse body mainly through urethral and esophageal system. The faster signal decrease of CPN6 treated mice organs further indicates its easier body clearance than that of CPN30. The fluorescence changes in blood for both CPN treated mice at varied time postadministration are shown in Figure 7B, CPN6 exhibits a much quicker signal decrease, which further demonstrates its faster body clearance, making it a noninvasive imaging nanoprobe.

## 3 Conclusion

In summary, we report a facile strategy to fabricate CPNs with sub‐10 nm size. Possessing such an ultrasmall size, high brightness in FR/NIR region, and blocked nonspecific cellular uptake, we demonstrate the superior performance of cRGD functionalized cRGD‐CPN6 for targeted imaging of cancer cells with integrin overexpression in a cell mixture. CPN6 shows a much higher fluorescence quantum yield (≈41%) as compared to CPN30 (≈25%). Due to its largely suppressed nonspecific cellular internalization, functionalized cRGD‐CPN6 shows excellent selective staining of MDA‐MB‐231 cells over controlled cells in cell mixture. Moreover, in vivo imaging and biodistribution experiments revealed faster body clearance of CPN6 relative to CPN30. Considering the extensive utilization of fluorescent NPs for in vitro and in vivo applications, this study provides a general strategy to fabricate NPs with high brightness and specificity, which opens new opportunities for immunostaining with high sensitivity.

## 4 Experimental Section

*Materials*: DSPE‐PEG_2000_‐Mal was purchased from Avanti Polar Lipids, Inc. QD655 was purchased from Life Technologies, Invitrogen, Singapore. Thiol (SH) modified cyclic arginine‐glycine‐aspartic acid (cRGD‐SH) was provided by GenicBio, China. All others chemicals were purchased from Sigma‐Aldrich.

*Characterization*: Shimadzu UV‐1700 and Edinburg FS5 spectrometer were used to measure UV–vis and photoluminescence spectra respectively. Zetasizer Nano S (Malvern Pte Ltd) was used to access the hydrodynamic sizes and zeta potentials. HR‐TEM (JEM‐2010F, JEOL, Japan) was used to study sample morphology. Fluorescence lifetime was measured following our previous experiment.^24^ A Nikon ECLIPSE Ti‐U inverted microscope with light source of a CW multiline Ar ion laser (Melles Griot, CA, USA) was used for single particle fluorescence measurement.^36^

*Fabrication of CPNs*: CPN30 was fabricated using a modified nanoprecipitation method according to literature. To synthesize CPN30, 1 mL of THF mixture of PFBTDBT (1 mg) and DSPE‐PEG‐Mal (2 mg) was added into 10 mL of MilliQ water under ultrasound sonication at 12 W output (XL2000, Misonix Incorporated, NY). THF was removed by placing the mixture in dark in fume hood under stirring at 600 rpm overnight. To synthesize CPN6, 1 mL of THF solution of PFBTDBT (0.1 mg mL^−1^) was added into 9 mL of aqueous solution containing DSPE‐PEG‐Mal (1 mg mL^−1^) under water batch sonication (S40, Elma Schmidbauer GmbH). After prolonged sonication of 30 min, the mixture was dialyzed against water using membrane with molecular cutoff of 6000–8000 Da to remove THF and excess DSPE‐PEG‐Mal polymers, and the NPs were collected for further use. To synthesize cRGD‐CPNs, excess amount of cRGD‐SH was added into CPN6 solution. After 4 h reaction, the product was dialyzed against water using membrane with molecular cutoff of 6–8 kDa to remove the excess of cRGD‐SH. The concentration of cRGD on CPN6 surface is determined by using Pierce Quantitative Peptide Assay (Life Technology Pte. Ltd.).

*Cell Culture*: HeLa cancer cells, breast cancer MDA‐MB‐231 cells, and normal fibroblast NIH‐3T3 cells (ATCC, USA) were cultured in Dulbecco's modified Eagle's medium (DMEM) containing 10% fetal bovine serum (FBS) and 1% penicillin/streptomycin solution, at 37 °C under humidified air containing 5% CO_2_.

*Flow Cytometry and Cell Imaging*: MDA‐MB‐231 cells were cultured in six‐well plate (Costar, IL, USA) at 37 °C. Upon reaching confluence, the culture medium was replaced with CPN6 or CPN30 suspended in FBS‐free DMEM (0.01 mg mL^−1^ based on PFBTDBT). After varied incubation time (1, 2, 4, and 6 h), the medium was removed, and the cells were washed with 1× PBS buffer and detached by 1× trypsin. The flow cytometry was measured using Cyan‐LX (DakoCytomation) and the histogram of each sample was obtained by counting 10 000 events. For confocal imaging, MDA‐MB‐231, HeLa, and NIH‐3T3 cells were cultured in separated wells of eight‐well chamber, the cells were incubated with unfunctionalized CPN6 or CPN30, or cRGD‐CPN6 (0.01 mg mL^−1^ based on PFBTDBT). After 6 h incubation, the cells were washed with 1× PBS buffer, incubated with Hoechst 33342 (Thermo Fisher Scientific Inc.) for 30 min, and imaged by CLSM (Zeiss LSM 410, Jena, Germany) with imaging software (Fluoview FV1000). For targeted imaging in the presence of multiple cell lines, Hela cells were pretreated with 2 × 10^−9^ m CellTracker540 (LunimiCell, Singapore) for 2 h. The treated Hela cells were then detached by 1× trypsin, centrifuged at 2000 rpm for 5 min, and then suspended in DMEM. Same amount of blank MDA‐MB‐231 cells suspension in DMEM was then mixed with CellTracker540 labeled HeLa cells together and further cultured overnight. The mixed cell monolayer was then incubated with cRGD‐CPN6 (0.01 mg mL^−1^ based on PFBTDBT) for 6 h. After discarding the culture medium, the cells were washed twice with 1× PBS buffer and imaged by CLSM. The green signal from CellTracker540 was collected between 505 and 525 nm upon excitation at 540 nm, the red fluorescence of cRGD‐CPN6 was collected above 650 nm upon excitation at 543 nm.

*Cell Uptake Mechanism*: MDA‐MB‐231 cells were cultured in separated wells of eight‐well chamber. The cells incubated with cRGD‐CPN6 (0.01 mg mL^−1^ based on PFBTDBT) for 6 h as control. For low temperature efficiency, cells were incubated with cRGD‐CPN6 (0.01 mg mL^−1^ based on PFBTDBT) for 6 h at 4 °C. For different endocytic mechanism study, the cells were pretreated with free cRGD (50 µg mL^−1^), chlorpromazine (10 µg mL^−1^), genistein (10 µg mL^−1^), nocodazole (5 µg mL^−1^), and LY294002 (20 µg mL^−1^) for 30 min at 37 °C, followed by incubation with cRGD‐CPN6 (0.01 mg mL^−1^ based on PFBTDBT) for 6 h. The cells were then washed three times with 1× PBS buffer prior to confocal imaging. The fluorescence intensity was analysis by Image J. Statistical analysis of the mean fluorescence intensity for each group of cells was compared with the control group to obtain the corresponding relative uptake efficiency. For free cRGD blocking experiments, the MDA‐MB‐231 cells were incubated with free cRGD (50 µg mL^−1^) for 30 min at 37 °C, followed by incubation with different CPNs (0.01 mg mL^−1^ based on PFBTDBT) for 6 h. The cells were then washed three times with 1× PBS buffer prior to confocal imaging. The fluorescence intensity were analysis by Image J. Statistical analysis of the mean fluorescence intensity for each group of cells was compared with the control group without free cRGD blocking to obtain the corresponding relative uptake efficiency

*Cytotoxicity of CPNs*: MTT bromide assay was used to evaluate the viabilities of MDA‐MB‐231 cells after CPN treatment. MDA‐MB‐231 cells were seeded in 96‐well plates (Costar, IL, USA) at an intensity of 5 × 10^4^ cells mL^−1^, respectively. After overnight culture, the cells were incubated with CPNs at varied concentrations (0.01, 0.05, 0.1 mg mL^−1^) for 48 h. After which, the cells were washed with 1× PBS buffer and incubated with MTT solution (0.5 mg mL^−1^, 100 µL per well). After 3 h incubation and discarding MTT solution, filtered DMSO (100 µL per well) was added to dissolve all the precipitates formed. The absorbance of MTT at 570 nm was monitored by the microplate reader (Genios Tecan), which was used to access the cell viability. The cells only incubated with culture medium were defined to have 100% viability.

*Body Clearance*: Healthy male Balb/c (InVivos Pte Ltd., Singapore) were used to access the in vivo body distribution and clearance of CPN30 and CPN6. All experimental procedures used in this study were approved by the Institutional Animal Care and Use Committee of the National University of Singapore. Mice were randomly assigned to two groups and each group contained eight mice. CPN30 and CPN6 (150 µL per mouse, at PFBTDBT mass concentration of 0.2 mg mL^−1^) were intravenously injected into each mouse in two groups respectively. At designated time point, two mice in each groups were sacrificed and the blood was collected through cardiac puncture. After coagulation at 4 °C, the blood was centrifuged at 10 000 rpm for 10 min. The fluorescence intensity of serum at different time point was measured by microplate reader (Genios Tecan). Moreover, after the mice in two groups were sacrificed, the normal organs of mice various tissues including intestine, liver, kidney, lung, spleen stomach, and heart were isolated and imaged using Xenogen IVIS Lumina II system for ex vivo fluorescence imaging.

## Supporting information

Supplementary
