# Supplementary material for: Ultrasmall Conjugated Polymer Nanoparticles with High Specificity for Targeted Cancer Cell Imaging
Source: Adv Sci (Weinh). 2017 May 1;4(9):1600407. doi: 10.1002/advs.201600407 (PMC5604381; doi:10.1002/advs.201600407)
Supplement: Supplementary file 1 — Supplementary [file ADVS-4-na-s001.pdf]

Copyright WILEY-VCH Verlag GmbH & Co. KGaA, 69469 Weinheim, Germany, 2016.

ADVANCED SCIENCE
Open Access

Supporting Information

for *Adv. Sci.,* DOI: 10.1002/advs.201600407

Ultrasmall Conjugated Polymer Nanoparticles with High Specificity for Targeted Cancer Cell Imaging

*Guangxue Feng, Jie Liu, Rongrong Liu, Duo Mao, Nikodem Tomczak, and Bin Liu**

Copyright WILEY-VCH Verlag GmbH & Co. KGaA, 69469 Weinheim, Germany, 2013.

Supporting Information

Ultra-Small Conjugated Polymer Nanoparticles with High Specificity for Targeted Cancer Cell Imaging

*Guangxue Feng, Jie Liu, Rongrong Liu, Duo Mao, Nikodem Tomczak and Bin Liu**

Keywords: Conjugated Polymers, organic nanoparticles, fluorescence imaging, cancer targeting

Table S1. Emission decay components of CPN6 and CPN30.

Sample	τ_1 (ns)	A_1(%)	τ_2 (ns)	A_2(%)	τ_{avg} (ns)	χ^2
CPN6	1.33	90.63	3.52	9.37	1.53	0.999
CPN30	1.30	99.14	9.75	0.86	1.38	0.998

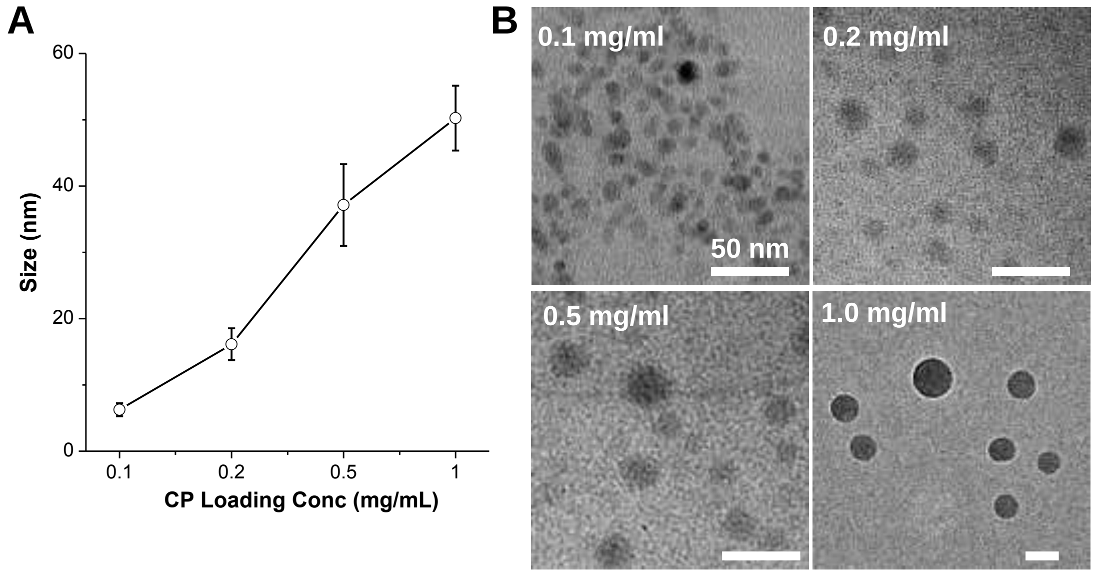

Figure S1. A) Size changes of CPNs upon increasing CP loading concentration while keeping other conditions unchanged, B) TEM images of CPNs fabricated from different CP loading concentrations, the size of scale bar is 50 nm.

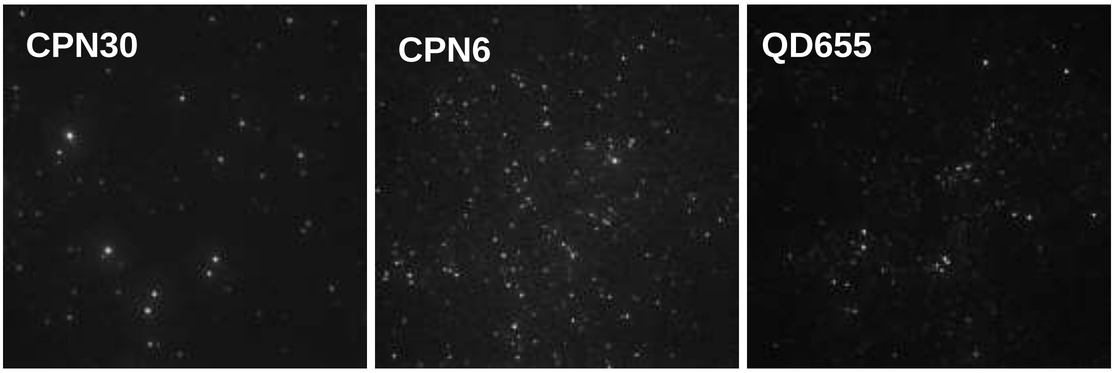

Figure S2 White field images of CPN30, CPN6, and QD655 under 488 nm laser excitation.

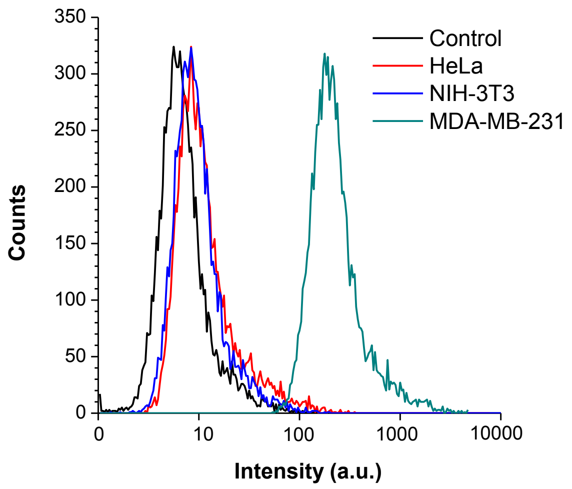

Figure S3. Flow cytometry histograms of HeLa, NIH-3T3, and MDA-MB-231 cells after 6 h incubation with cRGD-CPN6. The control is MDA-MB-231 cells without any treatment.

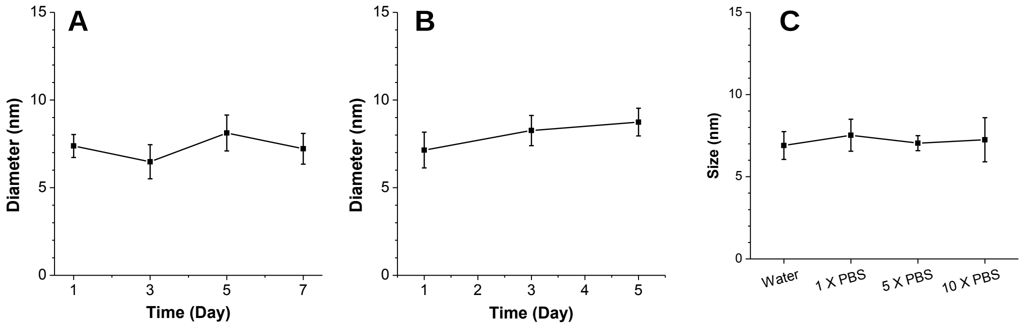

Figure S4. Size changes of CPN6 upon incubation in A) 1 × PBS buffer, or B) serum solution for varied time, and C) in PBS buffer with varied salt concentrations for 24 h.

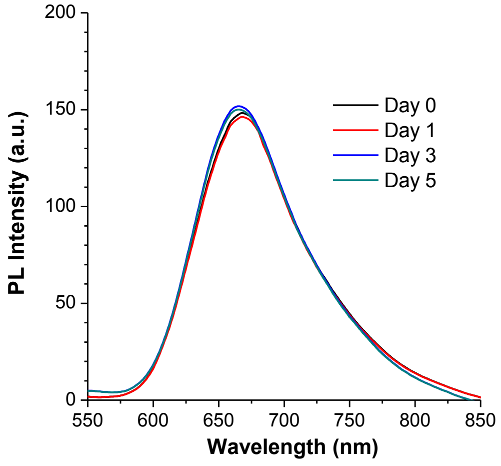

Figure S5. Fluorescence spectrum changes of CPN6 upon continuous incubation in 1× PBS buffer for 5 days.

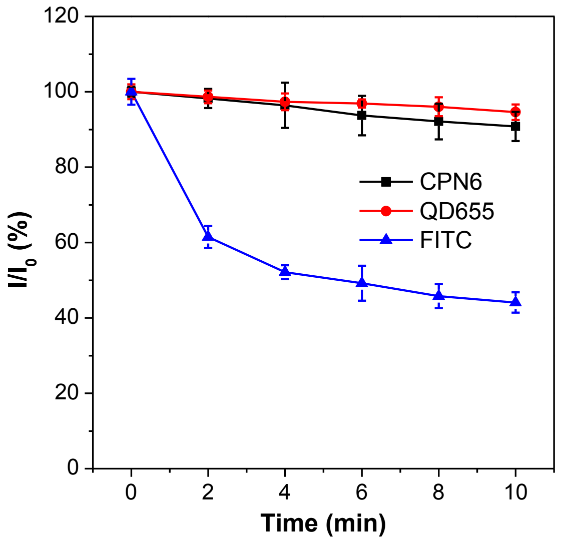

Figure S6. Photostability of CPN6, QD655, and FITC under continuous laser excitation. I_0 is the initial fluorescence intensity and I is the fluorescence intensity of each sample at various time points.

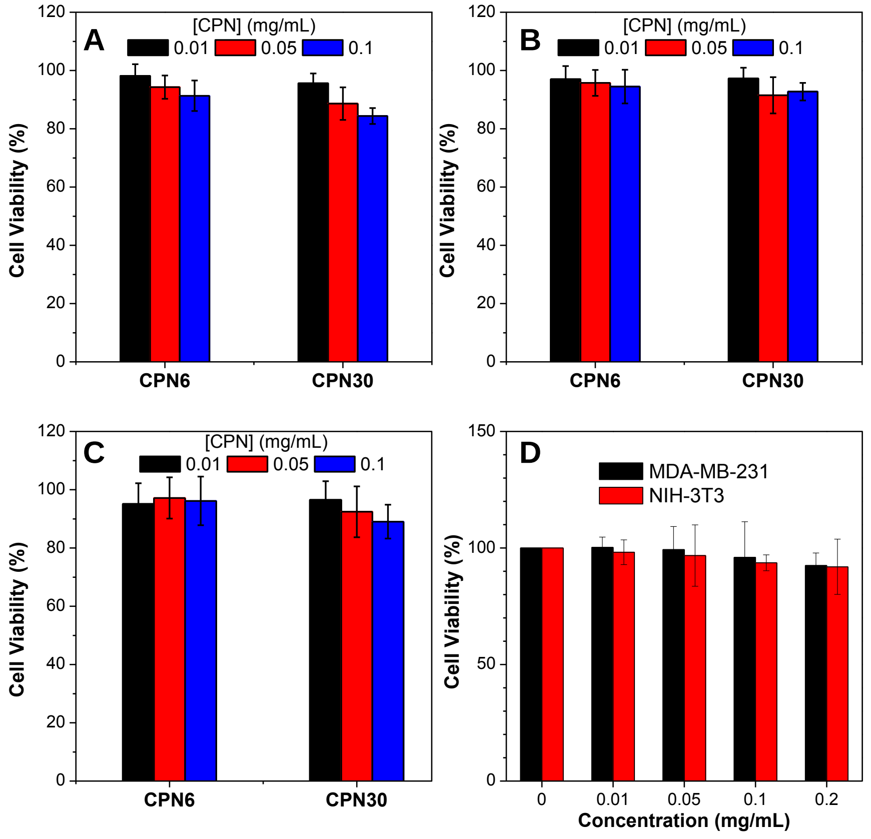

Figure S7. Cell viabilities of A) MDA-MB-231, B) HeLa, and C) NIH-3T3 cells after incubation with CPN6 or CPN30 at varied concentrations for 48 h, respectively. D) Cell viabilities of MDA-MB-231 and NIH-3T3 cells after incubation with cRGD-CPN6 at varied concentrations for 48 h, respectively.
